# Secondary Amplifier Sampling Component Design of an X-ray Framing Detector Based on a Streak Tube

**DOI:** 10.3390/s23052700

**Published:** 2023-03-01

**Authors:** Jing-jin Zhang, Yu-wei Xu, Fang-ke Zong, Li-hong Niu, Bao-guo Lei, Qin-lao Yang, Hou-zhi Cai

**Affiliations:** 1 Key Laboratory of Optoelectronic Devices and Systems of Ministry of Education and Guangdong Province, Shenzhen 518060, China; 2 College of Physics and Optoelectronic Engineering, Shenzhen University, Shenzhen 518060, China

**Keywords:** X-ray, high spatio-temporal resolution, streak tube, framing detector

## Abstract

The development of inertial confinement fusion (ICF) experiments necessitates the diagnostic instrument to have multiple frames with a high spatial and temporal resolution for the two-dimensional detection of the hot spot at the implosion end of the ICF. The existing sampling two-dimensional imaging technology in the world has superior performance; however, its subsequent development requires a streak tube with large lateral magnification. In this work, an electron beam separation device was designed and developed for the first time. The device can be used without changing the structure of the streak tube. It can be combined directly with the corresponding device and matched with a special control circuit. Based on the original transverse magnification, 1.77 times the secondary amplification can be achieved, which is conducive to expanding the recording range of the technology. The experimental results showed that the static spatial resolution of the streak tube after the inclusion of the device can still reach 10 lp/mm.

## 1. Introduction

Nuclear energy is one of the most promising future clean energy sources for mankind, owing to its rich energy resources and safe characteristics, and it can be generated from inertial confinement fusion (ICF) [1,2,3,4,5,6,7,8,9,10,11,12,13,14]. The ICF experiment depends on the hot spot of high-temperature and high-density material corresponding to the moment when the implosion compression reaches the maximum, and the state of the hot spot, namely the temperature and density, is an important criterion for fusion ignition [15]. Since the duration of the hot spot is approximately 100 ps, diagnostic equipment is required to provide two-dimensional (2D) spatial information and a temporal resolution of less than 30 ps. In addition, the diagnostic instrument should achieve high frame capture capability in the process of single-shot diagnosis to reduce the economic cost of the ICF experiment.

The transit time dispersion of electrons in the MCP (Micro Channel Plate) is large, which limits further improvement in the temporal resolution of the framing camera. The temporal resolution of the traditional MCP gated framing camera is only 60 ps [16,17,18]. Although the temporal resolution of the temporal broadened framing camera based on pulse dilation technology can be improved to 4 ps [19,20,21,22], the difference between the centre (10 lp/mm) and the edge resolution (5 lp/mm) is very large owing to the influence of the magnetic lens. The X-ray travelling wave gated and Kirkpatrick–Baez mirror framing imaging technology can reach a temporal resolution of 4 ps and a spatial resolution of 5 μm (100 lp/mm). However, owing to the limitation of the KB mirror structure, only four framing experimental results can be achieved at present [23].

The streak camera is diagnostic equipment that can simultaneously offer high spatial and temporal resolution. Its temporal resolution can range from picoseconds to femtoseconds while retaining high spatial resolution (better than 15 lp/mm). The spatial resolution of the single line of sight 2D framing technology realized by the LLNL laboratory, USA, using a one-dimensional streak camera can reach 20 lp/mm. Unfortunately, its temporal resolution is limited by the MCP gating technology; therefore, it still cannot meet the high index requirements of the multi-parameter ICF experiment. In 2017, a Japanese research team proposed a 2D Sampling Image X-ray Streak Camera (2D-SIXS) technology [24], which can achieve a spatial resolution of 20 μm (25 lp/mm) and a temporal resolution of 25 ps. This technology is based on the technique proposed by Niu [25], where a streak tube of large lateral magnification is required.

In this work, an electron beam separation device was designed and developed for the first time. Without changing the structure of the streak tube, the device can be used directly with the corresponding device. Based on the original transverse magnification, a secondary amplification of 1.77 times was obtained, which is conducive to expanding the recording range of the technology. The experimental results showed that the static spatial resolution of the streak tube combined with the device can still reach 10 lp/mm, which proved to be an extremely effective and convenient image device.

The content of this work is arranged as follows: the first part is about the research background, the second part is about the design concept of the designed secondary amplifier device, the third part is about the development of the corresponding physical device, the fourth part is about the experimental test and data analysis, and the fifth part is about the discussion of the results.

## 2. Design Strategy

**Figure 1:**
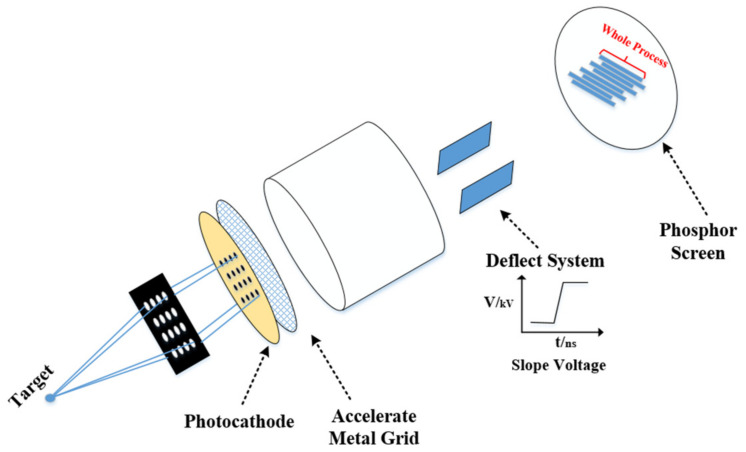
Working principle of 2D sampling streak camera: target is the hot spot in the fusion process; deflect system is composed of a pair of metal electrodes.

A 2D sampling image X-ray Streak camera includes an imaging hole array, a photocathode, an acceleration grid, a focusing electrode, a deflection system, and a fluorescent screen, as shown in Figure 1. The working principle is as follows: the incident light signal irradiates the imaging pinhole array for separation and sampling, then it falls on the photocathode and is converted into an optoelectronic beam, the beam is accelerated by the accelerating grid into the focus area for focusing, subsequently the timing information is mapped into space information by the deflection system, finally the phosphor screen is hit, and the image is read out via the CCD.

To obtain the whole process of ICF implosion with a better recording range, a larger scanning distance is preferred for pinhole imaging. The scanning distance R of the small hole image can be expressed as [25]

(1) $$R=MD\sqrt{1+{\left(MD/s\right)}^{2}}$$

where  D and d are the period intervals of the sampling pinhole array and the size of the pinhole, respectively, M represents the magnification, and $\;s=\sqrt{{\left(Md\right)}^{2}+\sigma^{-2}}$, where  σ is the spatial resolution of the streak camera.

From Equation (1), the scanning distance R is directly proportional to the quadratic power of the lateral magnification M of the streak camera. Therefore, the larger the lateral magnification of the streak tube is, the wider is the corresponding range.

In the electro-optical system, the trajectory of the electron beam cannot change suddenly; therefore, the secondary amplification device should ensure a continuous change in the electron beam trajectory. Since the cohesion of the electron beam in the focus area is related to its off-axis height (the direction of its movement), the secondary amplification device cannot be placed in the focus area; otherwise, the overall focus state will be affected. Therefore, the device can only be placed in the drift region.

**Figure 2:**
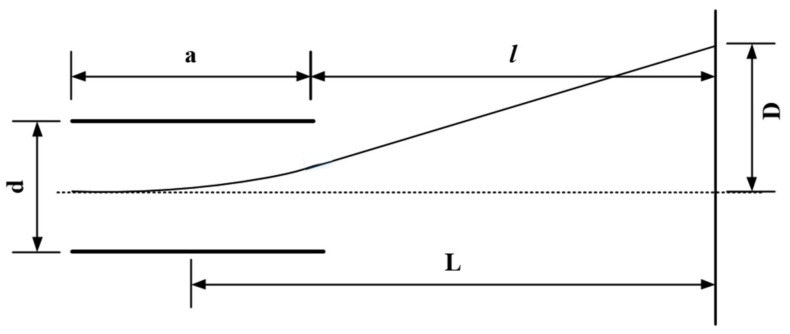
Structure of plate deflection plate: **d** is the plate spacing, **a** is the length of the plate, and **L** is the distance from the centre of the deflection electrode to the fluorescent screen.

Figure 2 shows the role of the deflection system in the continuous shifting of the electron beam trajectory in the drift region.

Consider a uniform field strength between plates ${\;E}_{y}=-\Delta V/d$, where  ΔV is the potential difference between two plates and d is the plate spacing. If the electrons moving at a speed $\nu_{0}=\sqrt{\frac{2e}{m}U_{a}}$ along the direction of the flat electrode are injected with the transverse electric field, then the deflection displacement P of the electron beam on the fluorescent screen can be obtained from the equation of motion of the electron:

(2) $$P=\frac{1}{2}\frac{V}{U_{a}}\frac{a}{d}\left(\frac{a}{2}+l\right)=\frac{aL}{2U_{a}d}V$$

where l is the distance from the deflection plate outlet to the fluorescent screen,  a is the length of the deflection plate, and $L=\frac{a}{2}+l$ is the distance from the deflection plate center to the fluorescent screen.

The final lateral magnification will be

(3) $$M=\frac{M_{o}h+P}{h}=M_{o}+\frac{P}{h},$$

where h is the off-axis height of the object point electron beam, and $M_{o}$ is the original lateral magnification.

## 3. Development and Experimental Testing of Device

**Figure 3:**
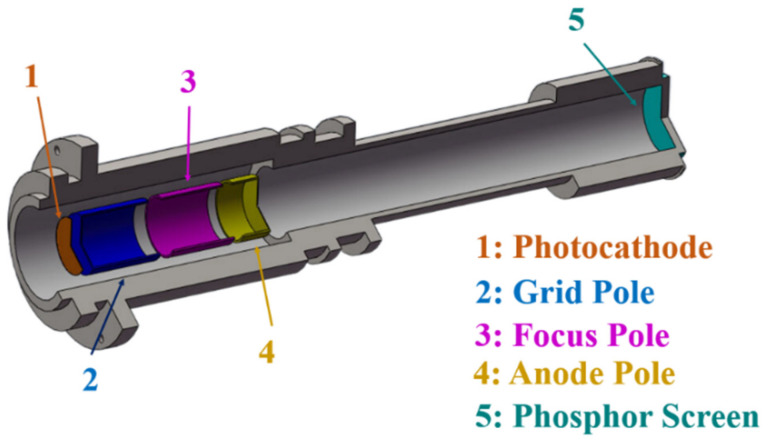
Structural diagram of streak tube.

To verify the secondary amplification effect of this device, an open X-ray streak tube with a known structure was selected. Its internal structure is shown in Figure 3, and the corresponding original parameters are listed in Table 1. 

**Figure 4:**
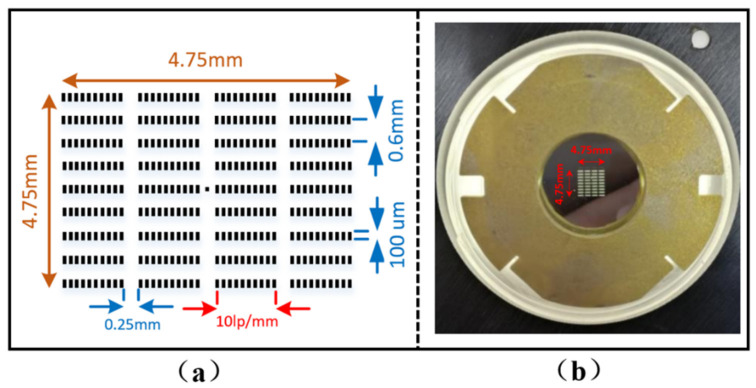
Structural diagram and physical drawing of photocathode: (**a**) schematic diagram of reticle structure; (**b**) physical drawing of photocathode.

Given the CCD area, the laboratory was 27 mm × 27 mm (2048 pixels × 2048 pixels); the maximum size of the image was limited to 27 mm. According to Table 1, the magnification of the streak tube was $\;M_{o}=2.84$; therefore, the maximum size of the image should not exceed 9.54 mm. To make full use of the object surface, the overall dimension was set to 4.75 mm × 4.75 mm. In this range, 9 lines × 4 slits with a width of 100 μm and a length of 1 mm were designed. The spacing between the stripes in each line was 0.6 mm, and the spacing between slits was 0.25 mm. The corresponding spatial resolution was 10 lp/mm. The specific structural parameters are shown in Figure 4a, where the central dot of the image is the central marking point.

For the development of the photocathode, first, the metal chromium reticle was processed and manufactured using an outsourcing method by Shen zhen Lu wei Optoelectronics Co., Ltd. (1/F, Block D, Huahan Innovation Park, No. 16, Langshan Road, North District, High-tech Zone, Nanshan District, Shenzhen, Guangdong Province, China) [26]. The reticle was used to generate the corresponding pattern on an aluminium film substrate through photolithography. Then, C8H8 was coated on the pattern to form a supporting film, and subsequently an 80 nm thick Au coating was formed by electron beam evaporation. The developed photocathode is shown in Figure 4b.

**Figure 5:**
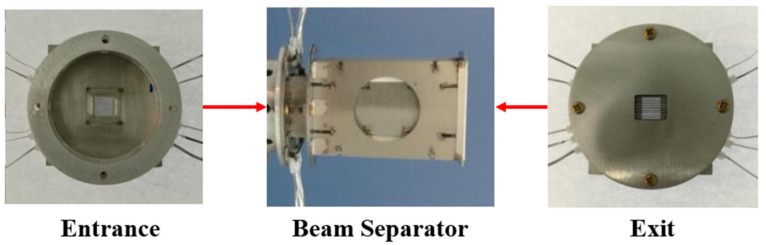
Physical drawing of separator: **the left section** is the entrance of beam separator, **the centre part** is the whole beam separator, and **the right section** is the shape of exit side.(The arrow in the figure is used to mark the position).

To facilitate the measurement, the secondary amplifier was placed in the drift zone where the magnification was 1 (the corresponding position is 260 mm away from the cathode through numerical calculation and located in the drift zone). Further, because the photocathode reticle had nine slits, the secondary amplification device was also made of nine pairs of deflector plates. The off-axis height of the electron beam at the entrance of each slit corresponded to the off-axis height, as shown in Figure 4. The parameters of each pair of deflector plates were set as follows: d=0.6 mm, a=8 mm, and plate width w=20 mm. The deflector plate was made of stainless-steel sheet. The final developed separator component is shown in Figure 5.

## 4. Experimental Test and Analysis

The separator was designed to realise the secondary amplification of the electron beam. It is necessary that, in each channel array of the separator device, the central channel array should keep the electron beam passing along the axis, while the other channels (non-centre channel array) must amplify in equal proportion, and the channels on both sides of the centre should have the same direction of secondary amplification of the electron beam before it is incident on the separator.

Therefore, the power-on mode of the separator shall meet the following conditions:

(1) From Formula (2), the corresponding voltage of the central channel array should be $\;V_{center}=0$.
(2) The voltage of each channel array above the central channel array should be $\;V_{Up}>0$, while the voltage of each channel array below should be $\;V_{down}<0$.
(3) The final amplification effect should be the same scale on both sides; therefore, $\left|V_{Up}\right|=\left|V_{down}\right|$.
(4) Similarly (from Figure 4a), the off-axis height of each row of stripes on both sides of the centre should be the integral multiple number of the base $\;h_{0}=0.6\;\mathrm{mm}$, that is, h_ I = I ∗ h_ 0 (i = −4, −3, …, 0, …, +3, +4) to achieve the same scale amplification on both sides, and according to Equation (2) there should be $P_{i}=i*P_{0}\;\left(i=-4,-3,\cdots 0,\cdots ,+3,+4\right)$, where $\;D_{0}$ is calculated by substituting $h_{0}$ into Equation (2).

**Figure 6:**
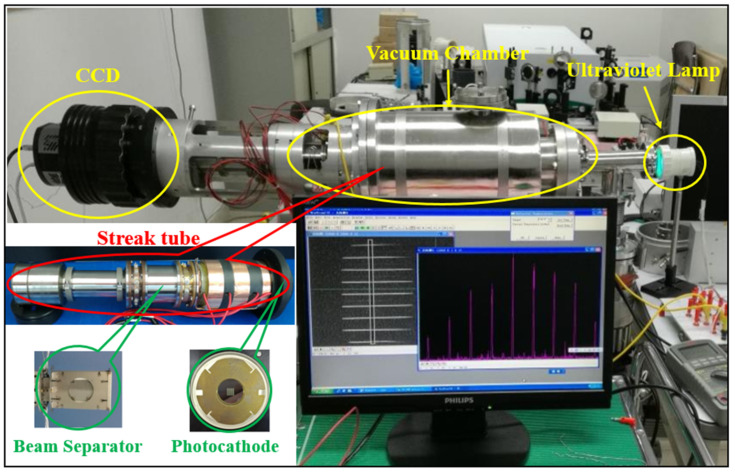
Streak tube and devices used for testing.

The existing streak tube used in the experiment is shown in Figure 6. Since only the spatial resolution and transverse magnification after the addition of the separator needed to be tested, only the ultraviolet lamp, scientific CCD (27 mm × 27 mm; 2048 pixel × 2048 pixel, pixel size corresponding to 13.2 μm) of Princeton Instruments, and a desktop computer was used for the test.

**Figure 7:**
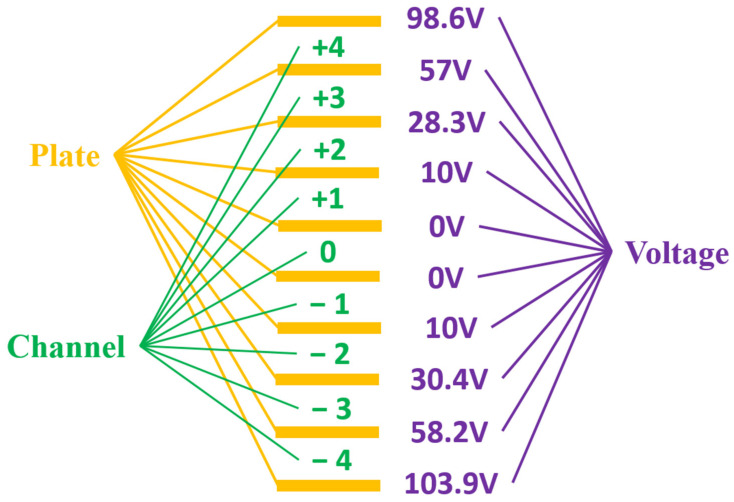
On board voltage of each channel array on the separator: **the yellow part** refers to the electrode corresponding to each channel of the separator, **the green part** refers to each channel in the separator, and **the purple part** refers to the DC voltage applied on each channel electrode.

To compare the secondary amplification effect of the separator, it was necessary to first test the image of the separator in power-off mode, to calibrate its intrinsic transverse magnification, and then in the power-on mode to assess the image for comparison. The voltage of each electrode in the streak tube during the test is shown in Table 2. After several experiments and debugging, the board voltage of each channel array on the separator was finally obtained, as shown in Figure 7.

**Figure 8:**
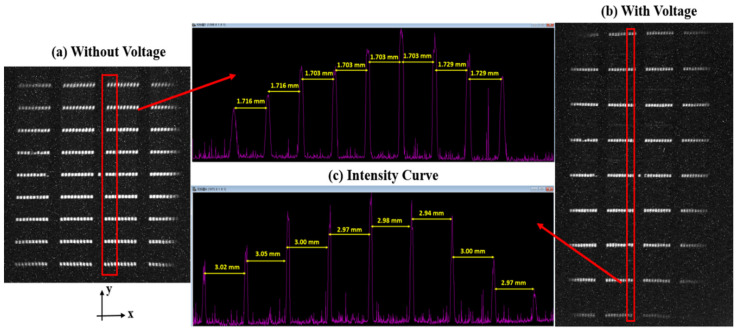
Test results: (**a**) the image obtained when the separator is not powered on; (**b**) the image obtained when the separator is powered on; (**c**) the distribution of the average value of intensity along the horizontal direction of the selected part in the red box in images (**a**,**b**) (the abscissa is the pixel position, and the ordinate is the intensity value).

Figure 8a,b show the streak patterns obtained before and after the power to the separator, respectively. Figure 8c displays the intensity value of each position obtained by averaging the x-direction value of the selected part of the red box in Figure 8a,b, with the intensity curve obtained along the y-direction. The abscissa is the pixel coordinate (corresponding to the y-direction in the red box). By reading the difference in the pixel coordinates corresponding to the peak spacing, the distance between each row of stripe images can be obtained by multiplying it by the pixel size (unit: mm). It can be seen from Figure 4a that the distance between the original row of stripes on the object surface was 0.6 mm. Therefore, the lateral magnification of the streak tube can be obtained when the separator is not powered, $\;M_{o}=1.7\mathrm{mm}/0.6\mathrm{mm}\approx 2.84$, and the transverse magnification of the streak tube can be obtained after the separator is powered,$\;M_{v}=3\;\mathrm{mm}/0.6\;\mathrm{mm}\approx 5$. The average secondary magnification ratio of the separator was $M_{sec}=M_{v}/M_{o}\approx 1.77$.

In addition, although the contrast of the fringes collected by the CCD edge was slightly poor after the separator was powered, the spatial resolution after the inclusion of the separator was at least 10 lp/mm (Figure 8) and the data of limit spatial resolution can be found in Table 3. 

The corresponding voltage differences of each channel array can be obtained from Figure 7, the total anode voltage value (Table 1), and by substituting the values  d=0.6 mm, a=8 mm, and plate width w=20 mm in Equations (2) and (3). The calculated results are listed in Table 4. 

It can be seen from Table 4 that the calculated results of the voltage difference obtained from the experimental test differed significantly for both channel sides. The main reason is that the internal electrodes of the streak tube were not strictly coaxial owing to the mounting inaccuracy; therefore, there was a radial shift in the direction of the internal electron beam. In addition, the object of the streak tube, i.e., the electron beam, is susceptible to the external magnetic field (including the geomagnetic field, the non-magnetic materials in the devices, etc.), which can increase the deflection of the electron beam, leading to deformation [27]. Further, the cohesion of the electron beam in the tube was proportional to the second power of the off-axis height [28]. Therefore, the electron beam was not symmetrical about the central (No. 0) channel array when it was incident on the separator. Therefore, the corresponding voltage value, the final lateral magnification, and the secondary magnification of each channel array are inconsistent. 

The imaging parameters obtained without and with the secondary amplification device powered on to the streak tube are shown in Table 5. It can be seen from Table 5 that the introduction of the device will not cause significant deterioration in the spatial resolution of the streak tube, and its geometric structure parameters will not change, but the final lateral magnification has increased by 1.77 times. From the analysis of Formula (1), one can find that the device will be very helpful for 2D Sampling Image X-ray Streak Camera (2D-SIXS) technology to expand the imaging range.

## 5. Conclusions

An electron beam separation device was designed and developed for 2D framing imaging technology. The device can be used directly with corresponding devices and special control circuits without changing the streak tube structure. Based on the original transverse magnification, a secondary amplification of 1.77 can be obtained, which is conducive to expanding the recording range of this technology. The experimental results showed that the static spatial resolution of the streak tube employed in this device can still reach 10 lp/mm.

The main performance index of the streak tube is its temporal resolution, which is closely related to the voltage slope of the deflection system, scanning speed, and other parameters, including the dynamic spatial resolution. The introduction of the secondary amplification device increased the size of the electron beam, which is not conducive to improving the deflection sensitivity. In this work, the power-on mode of the separator and its impact on the temporal-spatial resolution were not considered during the secondary amplification in the dynamic scanning of the streak tube. This part will be investigated further in a subsequent work.

## Figures and Tables

**Table 1:** The parameters of streak tube.

Length of Focus Region	Tube Length	Total Voltage	Lateral Magnification
135 mm	460 mm	10 kV	2.84

**Table 2:** Voltage of each electrode in streak tube experiment.

Pole	Photocathode	Grid	Focus Pole	Anode Pole
Voltage(kV)	−10	−5.967	−8.236	0

**Table 3:** The limit spatial resolution of images obtained with the separator powered on.

Channel	−4	−3	−2	−1	0	1	2	3	4
Original spatial resolution (lp/mm)	10								
Modulation degree (%)	0.514	0.622	0.611	0.693	0.649	0.595	0.545	0.511	0.475
Limit spatial resolution (lp/mm)	21.2	25.1	25	28.6	26.3	24	22.2	21.1	20.1

**Table 4:** Theoretical results.

Channel	−4	−3	−2	−1	0	+1	+2	+3	+4
ΔV (V)	−45.7	−27.8	−20.4	−10	0	10	18.3	28.3	41.6
P (mm)	−5.941	−3.614	−2.652	−1.3	0	1.3	2.379	3.679	5.408
$M_{v\_theo}$	5.30	4.84	5.04	5	—	5	4.81	4.87	5.08
$M_{sec\_theo}$	1.867	1.704	1.775	1.761	—	1.761	1.694	1.715	1.789

**Table 5:** Comparison of imaging performance of the streak tube without and with secondary amplification device powered on.

Parameters	Streak Tube with Secondary Amplification Device Powered on	Streak Tube without Secondary Amplification Device Powered on
Tube length (mm)	460	460
Diameter of tube (mm)	60	60
Static spatial resolution (lp/mm)	10	10
Transverse magnification	5.03	2.84

## Data Availability

Data underlying the results presented in this paper are not publicly available at this time but may be obtained from the authors via email upon reasonable request.
